# Extent of Tumor Resection and Survival in Pediatric Patients With High-Grade Gliomas: A Systematic Review and Meta-analysis

**DOI:** 10.1001/jamanetworkopen.2022.26551

**Published:** 2022-08-16

**Authors:** Rami Hatoum, Jia-Shu Chen, Pascal Lavergne, Nathan A. Shlobin, Andrew Wang, Lior M. Elkaim, Philippe Dodin, Charles P. Couturier, George M. Ibrahim, Aria Fallah, Dominic Venne, Sebastien Perreault, Anthony C. Wang, Nada Jabado, Roy W. R. Dudley, Alexander G. Weil

**Affiliations:** 1 University of Montréal School of Medicine, Montréal, Quebec, Canada; 2 The Warren Alpert Medical School of Brown University, Providence, Rhode Island; 3 Department of Neurological Surgery, University of Washington, Seattle; 4 Department of Neurological Surgery, Northwestern University Feinberg School of Medicine, Chicago, Illinois; 5 Department of Neurosurgery, David Geffen School of Medicine at UCLA (University of California, Los Angeles); 6 College of Medicine, Charles R. Drew University of Medicine and Science, Los Angeles, California; 7 Division of Neurology and Neurosurgery, McGill University, McGill University Health Center, Montreal, Quebec, Canada; 8 Medical Library, Centre Hospitalier Universitaire (CHU) Sainte-Justine Children’s, Montréal, Quebec, Canada; 9 Department of Neurology and Neurosurgery, Montréal Neurological Institute–Hospital, Montréal, Quebec, Canada; 10 Division of Neurosurgery, The Hospital for Sick Children, University of Toronto, Toronto, Ontario, Canada; 11 Department of Pediatrics, David Geffen School of Medicine at UCLA; 12 Division of Neurosurgery, Ste Justine Hospital, University of Montréal, Montréal, Quebec, Canada; 13 Department of Neurology, CHU Sainte-Justine, Montréal, Quebec, Canada; 14 Department of Human Genetics, McGill University, Montréal, Quebec, Canada; 15 Department of Pediatrics, McGill University and McGill University Health Centre Research Institute, Montréal, Quebec, Canada; 16 Neurosurgery Service, Department of Surgery, McGill University and McGill University Health Centre Research Institute, Montréal, Quebec, Canada; 17 Neurosurgery Service, Department of Surgery, University of Montreal Hospital Center, Montréal, Quebec, Canada; 18 Sainte-Justine University Hospital Research Center, Montréal, Quebec, Canada

## Abstract

**Importance:**

Pediatric patients with high-grade gliomas have a poor prognosis. The association among the extent of resection, tumor location, and survival in these patients remains unclear.

**Objective:**

To ascertain whether gross total resection (GTR) in hemispheric, midline, or infratentorial pediatric high-grade gliomas (pHGGs) is independently associated with survival differences compared with subtotal resection (STR) and biopsy at 1 year and 2 years after tumor resection.

**Data Sources:**

PubMed, EBMR, Embase, and MEDLINE were systematically reviewed from inception to June 3, 2022, using the keywords *high-grade glioma*, *pediatric*, and *surgery*. No period or language restrictions were applied.

**Study Selection:**

Randomized clinical trials and cohort studies of pHGGs that stratified patients by extent of resection and reported postoperative survival were included for study-level and individual patient data meta-analyses.

**Data Extraction and Synthesis:**

Study characteristics and mortality rates were extracted from each article. Relative risk ratios (RRs) were pooled using random-effects models. Individual patient data were evaluated using multivariate mixed-effects Cox proportional hazards regression modeling. The PRISMA reporting guideline was followed, and the study was registered a priori.

**Main Outcomes and Measures:**

Hazard ratios (HRs) and RRs were extracted to indicate associations among extent of resection, 1-year and 2-year postoperative mortality, and overall survival.

**Results:**

A total of 37 studies with 1387 unique patients with pHGGs were included. In study-level meta-analysis, GTR had a lower mortality risk than STR at 1 year (RR, 0.69; 95% CI, 0.56-0.83; *P* < .001) and 2 years (RR, 0.74; 95% CI, 0.67-0.83; *P* < .001) after tumor resection. Subtotal resection was not associated with differential survival compared with biopsy at 1 year (RR, 0.82; 95% CI, 0.66-1.01; *P* = .07) but had decreased mortality risk at 2 years (RR, 0.89; 95% CI, 0.82-0.97; *P* = .01). The individual patient data meta-analysis of 27 articles included 427 patients (mean [SD] age at diagnosis, 9.3 [5.9] years), most of whom were boys (169 of 317 [53.3%]), had grade IV tumors (246 of 427 [57.7%]), and/or had tumors that were localized to either the cerebral hemispheres (133 of 349 [38.1%]) or midline structures (132 of 349 [37.8%]). In the multivariate Cox proportional hazards regression model, STR (HR, 1.91; 95% CI, 1.34-2.74; *P* < .001) and biopsy (HR, 2.10; 95% CI, 1.43-3.07; *P* < .001) had shortened overall survival compared with GTR but no survival differences between them (HR, 0.91; 95% CI, 0.67-1.24; *P* = .56). Gross total resection was associated with prolonged survival compared with STR for hemispheric (HR, 0.29; 95% CI, 0.15-0.54; *P* < .001) and infratentorial (HR, 0.44; 95% CI, 0.24-0.83; *P* = .01) tumors but not midline tumors (HR, 0.63; 95% CI, 0.34-1.19; *P* = .16).

**Conclusions and Relevance:**

Results of this study show that, among patients with pHGG, GTR is independently associated with better overall survival compared with STR and biopsy, especially among patients with hemispheric and infratentorial tumors, and support the pursuit of maximal safe resection in the treatment of pHGGs.

## Key Points

**Question:**

Is the extent of tumor resection associated with survival in pediatric patients with high-grade gliomas?

**Findings:**

In this systematic review and meta-analysis of 37 studies involving 1387 unique pediatric patients with high-grade gliomas, gross total resection was independently associated with better overall survival compared with subtotal resection and biopsy, especially in patients with hemispheric and infratentorial tumors.

**Meaning:**

Findings of this study suggest that maximal safe resection should be performed when feasible to treat high-grade gliomas in pediatric patients.

## Teaser

This systematic review and meta-analysis describes postoperative outcomes associated with gross total resection, subtotal resection, and biopsy in children with high-grade gliomas.

## Introduction

Malignant brain tumors are the leading cause of cancer death in children.^1^ High-grade gliomas (HGGs) are among the most lethal of cancers, with a median overall survival (OS) of 14 to 20 months after optimal multimodal therapy.^2,3,4^ The current standard of care for HGGs in adults includes maximal safe resection, high-dose radiotherapy, and chemotherapy.^5^

The survival benefit of gross total resection (GTR) compared with subtotal resection (STR) for HGGs in adults is well established.^6^ Current data suggest incremental improvements in both survival and function with a greater extent of resection (EOR).^7,8^ Although there is robust evidence supporting greater EOR in adults, evidence supporting greater EOR for HGGs in children is limited.^9^ Furthermore, pediatric HGGs (pHGGs) are distinct from adult HGGs in molecular signature as well as clinical and radiologic presentation.^10,11,12,13,14^ Thus, whether the evidence for EOR in adults translates to benefits for children remains to be seen.^14^ Current evidence of the implications of EOR for survival in pHGGs includes prospective data demonstrating an association between greater-than-90% tumor resection and improved progression-free survival (PFS).^2^ Other studies have found associations among EOR, OS, and PFS and identified GTR as an independent factor in improved survival.^15,16,17,18,19^

However, GTR in children is often challenging because of tumors in eloquent areas or midline locations (eg, thalamus and brainstem), the most prevalent anatomical sites for pHGGs.^9^ The survival benefit of GTR in midline locations has not been demonstrated. Furthermore, it is unclear whether STR, compared with biopsy, confers a survivorship advantage in pHGGs and should be aggressively pursued. As a result, clinicians currently face the challenge of balancing the uncertain potential benefits of aggressive tumor resection with the nonnegligible risks of postoperative neurologic impairment and morbidity.

The need for studies elucidating the association between EOR and survival in pHGGs is therefore emphasized. To address this need, we conducted a systematic review and meta-analysis of the published literature to ascertain whether GTR in hemispheric, midline, or infratentorial pHGGs is independently associated with survival differences compared with STR and biopsy at 1 year and 2 years after tumor resection. Ultimately, these findings are intended to help guide clinicians who treat children and youth with HGGs.

## Methods

We followed the Preferred Reporting Items for Systematic Reviews and Meta-analyses (PRISMA) reporting guideline and Meta-Analysis of Observational Studies in Epidemiology (MOOSE) checklist.^20,21^ A review protocol was registered a priori through the International Prospective Register of Systematic Reviews (PROSPERO) after preliminary searches were conducted.

### Eligibility Criteria

Randomized clinical trials (RCTs), cohort studies, and conference abstracts were included if the study population (1) was predominantly (>80%) younger than 21 years, the upper age limit of pediatric populations according to the American Academy of Pediatrics; (2) predominantly (>50%) had histologically confirmed intracranial HGG diagnoses (WHO [World Health Organization Classification of Tumors of the Central Nervous System] grade III-IV); (3) underwent surgical cytoreduction; (4) was stratified into 3 or more EOR categories (GTR, STR, or biopsy); and (5) reported objective data on OS and/or PFS. Studies were excluded if they did not contain EOR comparison groups or if they focused on diffuse intrinsic pontine glioma because it exhibits a distinct entity that is not amenable to tumor resection.

### Search Strategy and Selection Process

We developed the search strategy with a medical librarian trained in performing systematic reviews (P.D.), using the keywords *high-grade glioma*, *pediatric*, and *surgery* (eMethods in the Supplement). We searched PubMed, EBMR (Evidence-Based Medicine Reviews), Embase, and MEDLINE from inception to June 3, 2022. No period or language restrictions were applied. Non-English articles were translated using online tools. The reference lists of included articles were manually searched during full-text review to capture additional articles that met the inclusion criteria but were indexed discordantly from the search strategy. Study selection occurred in 2 stages and was performed by 2 of us (R.H., P.L.). First, we independently screened titles and abstracts to identify relevant studies. Second, we independently performed full-text reviews of the screened studies using a predetermined, pilot-tested form containing all of the eligibility criteria. Disagreements (κ = 0.92) were resolved through consultation with a predetermined third author (A.G.W.).

### Data Extraction

Two of us (R.H., J.-S.C.) extracted the data independently, and disagreements were resolved by discussion with a third author (A.G.W.). We defined EOR according to the authors of studies and summarized EOR as GTR, STR, or biopsy. Resection that was greater than 90% was considered to be GTR. Subtotal resection encompasses all forms of partial resection and is considered to be less-than-90% tumor removal if not explicitly defined.

For the study-level meta-analysis, the comparison of interest was OS at 1 year and 2 years after tumor resection. One-year OS was chosen because it approximates the median OS for pHGGs, thus making 2-year OS a clinically relevant comparison that only 10% to 30% of patients reach.^3,22^ Six-month and 1-year PFS were also collected because they represent the standard timeline of clinical progression.^3,22^ For studies that did not report outcomes in the text, relative risk ratios (RRs) at the aforementioned time points were extracted from their Kaplan-Meier curves using a Cochrane-approved pixel-coordinate method that maps axis intercepts and calculates the percentage of at-risk patients.^23^

Individual patient data (IPD) reported in included articles were collected to create a pooled cohort for the IPD meta-analysis (IPDMA). Authors of included articles were not contacted when IPD were missing or not reported. Extracted characteristics included EOR and OS and, if available, age at diagnosis, sex, tumor location (hemispheric, midline, or infratentorial), tumor histologic grade (WHO grade III, IV), and adjuvant treatments (radiotherapy with concomitant chemotherapy, radiotherapy only, chemotherapy only, or no adjuvant treatment). Tumors were considered to be midline if they affected supratentorial midline structures (notably, the thalamus), whereas tumors were considered to be infratentorial if they resided within the brainstem, cerebellum, or spinal cord.^24^

### Quality Appraisal

The quality of each article was graded independently by one of us (R.H.) using the Newcastle-Ottawa Scale for nonrandomized observational studies^25^ and the Cochrane Collaboration risk-of-bias tool for RCTs.^26^ The Newcastle-Ottawa Scale is a method for assessing risk of bias based on appropriate participant selection, exposure measures, outcome variables, and comparability; the summary score given to studies can be categorized as poor (0-3 points), fair (4-6 points), or good (≥7 points) quality (maximum score, 9 points). The Cochrane Collaboration risk-of-bias tool covers 7 methodological domains, including selection, performance, attrition, and reporting, that can be graded as having low, moderate, high, or unclear risk of bias.

### Statistical Analysis

Descriptive statistics were used to present the IPD, with continuous variables summarized using means and SDs and categorical variables described using frequencies and percentages. Continuous variables of each EOR subgroup were compared using 1-way analysis of variance with the post hoc Tukey test. Categorical variables were evaluated using χ^2^ tests. A multivariate mixed-effects Cox proportional hazards regression model using the included study as a random-effects variable to account for methodological and clinical variations across studies was constructed to identify independent factors in time to mortality. Variables were included in the multivariate model if they were associated with OS in the univariate analysis with *P* < .20. Hazard ratios (HRs), 95% CIs, and *P* values were reported for each covariate. Kaplan-Meier curves with log-rank test were constructed to visualize survival differences on a time-to-event basis. A 2-tailed *P* ≤ .05 was the threshold for statistical significance.

Aggregate data were pooled using random-effects models and the Mantel-Haenszel method to account for between-study variability. Pooled RRs of mortality and progression with 95% CIs were generated for the comparisons of interest at each time point. Heterogeneity among studies was assessed using the Cochran *Q* statistic to calculate the *I*^2^ statistic. *P* < .10 for Cochran *Q* and *I^2^*>50% were considered to be heterogeneous and warranted investigation to identify the source of heterogeneity.^27^ Publication bias was evaluated using funnel plots and the modified Egger test.^28,29^ Sensitivity analyses were performed to assess the robustness of the findings by removing subgroups of studies with potential bias that may distort results and then repeating pooling. The largest study based on the Surveillance, Epidemiology, and End Results (SEER) Program data was removed to gauge its role in changing pooled results. Results were also reassessed after excluding (1) studies that had RRs extracted from Kaplan-Meier curves and (2) cohort studies with a quality score lower than 6 points and RCTs with a high risk of bias, given their methodological limitations, to ascertain these studies’ impact. All analyses were performed in RStudio, version 1.2.1335 (RStudio, PBC).

## Results

### Study Selection

The search yielded 11 973 articles (eFigure 1 in the Supplement). After exclusion of 3120 duplicates, 8853 unique articles underwent title and abstract screening, of which 281 were full-text articles assessed for eligibility. Ultimately, 37 articles published between March 1984 and April 12, 2022, reporting on 1387 unique patients were included for data extraction.^15,16,18,19,30,31,32,33,34,35,36,37,38,39,40,41,42,43,44,45,46,47,48,49,50,51,52,53,54,55,56,57,58,59,60,61,62^ Of the 37 articles, 8 (21.6%) were identified from screening the reference lists of the 29 (78.4%) initially found through the search strategy.^31,32,36,40,49,56,58,59^ For the IPDMA, 27 articles (73.0%) provided IPD on 427 unique patients (mean [SD] age at diagnosis, 9.3 [5.9] years; 169 of 317 [53.3%] were boys, and 148 were girls [46.7%]).^18,30,31,32,33,35,36,37,38,40,41,42,43,44,45,49,50,52,53,54,55,56,57,58,59,60,62^ Twenty-six articles (70.3%) were observational cohort studies, and 10 (27.0%) were prospective studies. Most of the 36 nonrandomized observational studies (33 [91.7%]) were graded as fair or good quality, and the only RCT was graded as poor quality (eTables 1 and 2 in the Supplement). Eight articles did not explicitly report RRs of mortality (6 of 37 [16.2%]) or progression (8 of 37 [21.6%]) and had them extracted from their respective Kaplan-Meier curves.^16,34,39,46,47,48,51,61^

### Study-Level Meta-analysis

**Figure 1.**
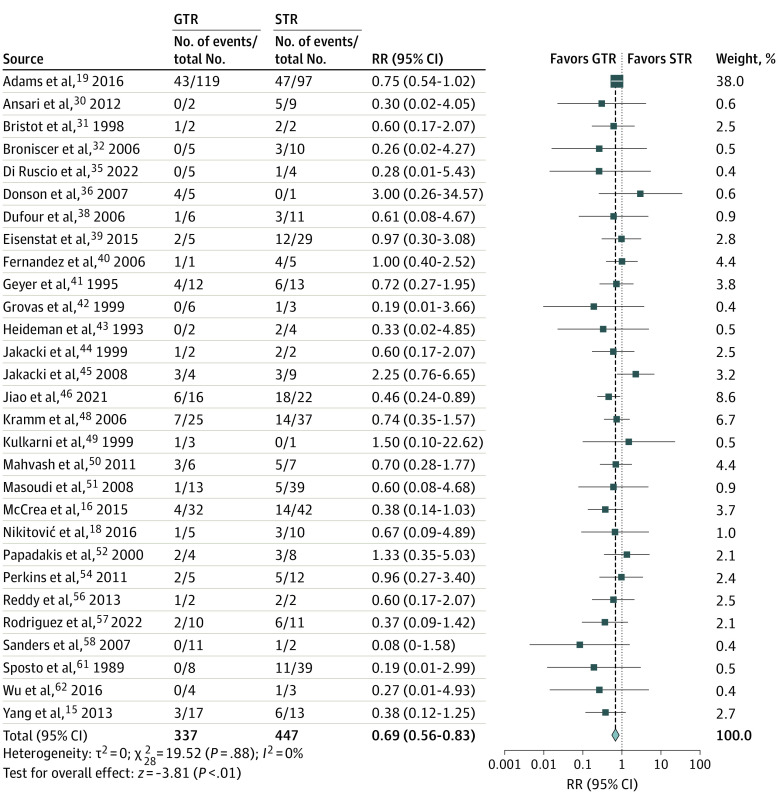
Forest Plot of Relative Risk (RR) for 1-Year Mortality for Gross Total Resection (GTR) vs Subtotal Resection (STR) Blue squares indicate weight-adjusted relative risk ratios (RRs) of individual studies; horizontal black lines, 95% CIs; and blue diamond, the pooled relative RR estimate.

**Figure 2.**
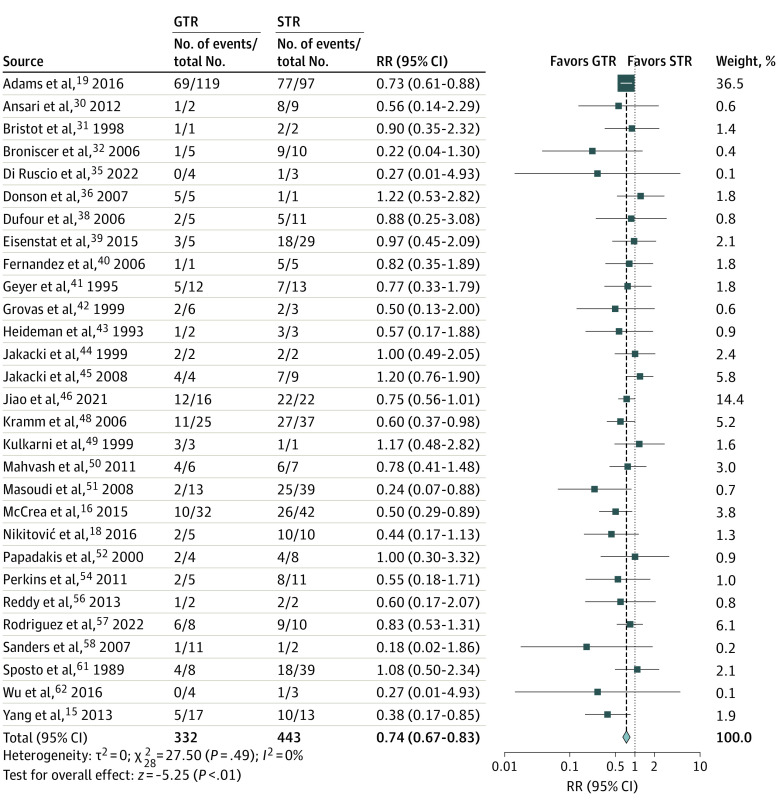
Forest Plot of Relative Risk (RR) for 2-Year Mortality for Gross Total Resection (GTR) vs Subtotal Resection (STR) Blue squares indicate weight-adjusted relative risk ratios (RRs) of individual studies; horizontal black lines, 95% CIs; and blue diamond, the pooled relative RR estimate.

Gross total resection was associated with lower mortality risk than STR at 1 year (RR, 0.69; 95% CI, 0.56-0.83; *P* < .001) and 2 years (RR, 0.74; 95% CI, 0.67-0.83; *P* < .001) after tumor resection (Figure 1 and Figure 2). When STR was compared with biopsy, no survival advantages were observed at 1 year (RR, 0.82; 95% CI, 0.66-1.01; *P* = .07), but the 2-year mortality was improved (RR, 0.89; 95% CI, 0.82-0.97; *P* = .01) (eFigure 2 in the Supplement). The association between EOR and PFS was similar, with GTR associated with decreased progression risk at 6 months (RR, 0.62; 95% CI, 0.46-0.82; *P* = .001) and 1 year (RR, 0.74; 95% CI, 0.60-0.90; *P* = .003) (eFigure 3 in the Supplement). When PFS between STR and biopsy was compared, there were no differences in progression risk at 6 months (RR, 0.91; 95% CI, 0.65-1.28; *P* = .59) or 1 year (RR, 1.00; 95% CI, 0.88-1.13; *P* = .95) (eFigure 4 in the Supplement). Heterogeneity across publications for OS comparisons between GTR and STR as well as STR and biopsy was not significant (for all comparisons, *Q* statistic *P* > .10; *I*^2^<50%).

Funnel plots were generated along with each survival forest plot to evaluate publication bias (eFigure 5 in the Supplement). The largest funnel plot comparing the 1-year OS between GTR and STR displayed asymmetry at the level of small studies, with a modest deficit in studies favoring STR over GTR. However, the modified Egger test did not demonstrate significant publication bias (–0.38; *P* = .14). During sensitivity analysis, removing SEER Program data did not change the significance of results. Excluding manually extracted outcomes from Kaplan-Meier curves and studies with lower-quality grades also did not affect the results between GTR and STR. However, the survival advantage associated with STR over biopsy at 2 years was lost (eTable 3 in the Supplement).

### IPD Meta-analysis

**Figure 3.**
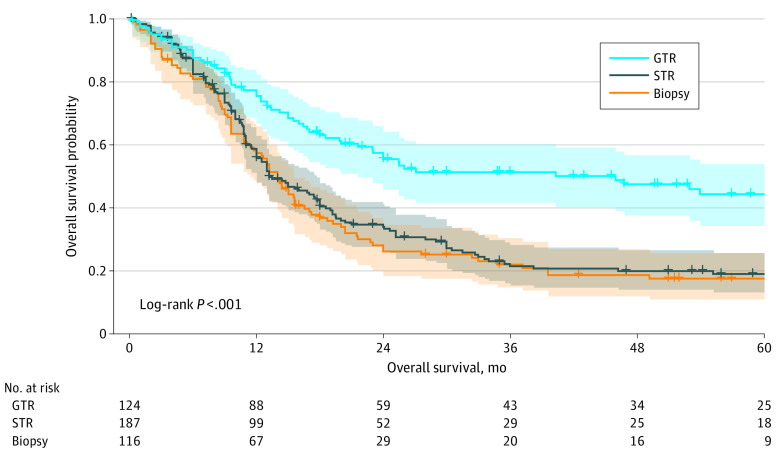
Kaplan-Meier Curve Comparing Overall Survival According to Extent of Resection GTR indicates gross total resection; STR, subtotal resection.

The cohort of patients with IPD (n = 427) was composed of predominantly boys (169 of 317 [53.3%]). Most patients had WHO grade IV tumors (246 of 427 [57.7%]) localized to the cerebral hemispheres (133 of 349 [38.1%]) or midline structures (132 of 349 [37.8%]). Characteristics of of patients with IPD are stratified by EOR and summarized in Table 1. The prevalence was 29.0% (n = 124) for GTR, 43.8% (n = 187) for STR, and 27.2% (n = 116) for biopsy. There were no differences in sex across the subgroups; however, there were significant differences in age at diagnosis, tumor location, tumor histologic grade, and adjuvant treatment (for all comparisons, *P* < .001). Patients with GTR were younger than those with STR (mean [SD] age, 7.3 [5.9] years vs 9.7 [5.7] years; *P* < .001) or biopsy (vs 10.8 [5.7] years; *P* < .001). The GTR cohort also had higher rates of infratentorial tumors, WHO grade IV tumors, and patients who did not receive both radiotherapy and chemotherapy. However, patients who underwent GTR had longer OS compared with survival of those who underwent STR and biopsy (27.1 months vs 13.2 months vs 14.0 months; log-rank *P* < .001) (Figure 3).

**Table 1.** Baseline Characteristics of 427 Patients in the Individual-Patient Data Meta-analysis

Characteristic	Extent of resection, No. (%)			*P* value
	GTR (n = 124)	STR (n = 187)	Biopsy (n = 116)	
				
Age at diagnosis, mean (SD), y	7.3 (5.9)	9.7 (5.7)	10.8 (5.7)	<.001^a^
Sex (n = 317)				
Female	35 (40.7)	67 (48.6)	46 (49.5)	.42
Male	51 (59.3)	71 (51.4)	47 (50.5)	
Tumor location (n = 349)				
Hemispheric	50 (46.3)	59 (39.9)	24 (25.8)	<.001^a^
Midline	23 (21.3)	58 (39.2)	51 (54.8)	
Infratentorial	35 (32.4)	31 (20.9)	18 (19.4)	
Tumor histologic grade (n = 427)				
WHO grade III	40 (32.3)	73 (39.0)	68 (58.6)	<.001^a^
WHO grade IV	84 (67.7)	114 (61.0)	48 (41.4)	
Adjuvant treatment (n = 421)				
Radiotherapy with chemotherapy	72 (59.0)	130 (70.7)	88 (76.5)	<.001^a^
Radiotherapy alone	7 (5.7)	22 (12.0)	12 (10.4)	
Chemotherapy alone	34 (27.9)	29 (15.8)	13 (11.3)	
None	9 (7.4)	3 (1.6)	2 (1.7)	
Overall survival, median (95% CI), mo^b^	27.1 (41.7-60.3)	13.2 (12.0-17.7)	14.0 (11.4-15.6)	<.001^a^

Abbreviations: GTR, gross total resection; STR, subtotal resection; WHO, World Health Organization Classification of Tumors of the Central Nervous System.

^a^ *P* < .05 indicates statistically significant difference in characteristics between groups.

^b^ Overall survival is reported as median (95% CI), both of which were derived from Kaplan-Meier analysis.

Univariate mixed-effects Cox proportional hazards regression modeling showed that STR (HR, 1.99; 95% CI, 1.45-2.72; *P* < .001) and biopsy (HR, 2.09; 95% CI, 1.48-2.95; *P* < .001) were associated with worse OS compared with GTR (Table 2). Midline and infratentorial tumors as well as radiotherapy alone and no adjuvant treatment were also associated with worse OS compared with hemispheric tumors and adjuvant radiotherapy with concomitant chemotherapy (eFigure 6 in the Supplement). The association between EOR and OS persisted during multivariate analysis after controlling for tumor location and adjuvant treatment. Subtotal resection (HR, 1.91; 95% CI, 1.34-2.74; *P* < .001) and biopsy (HR, 2.10; 95% CI, 1.43-3.07; *P* < .001) were independently associated with worse estimated outcome compared with GTR. However, STR had no associated survival advantage (HR, 0.91; 95% CI, 0.67-1.24; *P* = .56) over biopsy. Other independent associations included the association of midline (HR, 1.74; 95% CI, 1.16-2.63; *P* = .008) and infratentorial (HR, 2.21; 95% CI, 1.48-3.31; *P* < .001) tumors, radiotherapy alone (HR, 2.84; 95% CI, 1.67-4.81; *P* < .001), and no adjuvant treatment (HR, 4.93; 95% CI, 2.98-8.16; *P* < .001) with shortened survival.

**Table 2.** Univariate and Multivariate Mixed-Effects Cox Proportional Hazards Regression Model of Overall Survival for Patients in the Individual-Patient Data Meta-analysis

Factor	Univariate HR (95% CI)	*P* value	Multivariate HR (95% CI)	*P* value
Older publication year	1.01 (0.98-1.04)	.54	NA	NA
Older age at diagnosis	1.00 (0.99-1.01)	.31	NA	NA
Male sex	1.01 (0.78-1.32)	.92	NA	NA
Extent of resection				
GTR	1 [Reference]		1 [Reference]	
STR	1.99 (1.45-2.72)	<.001^a^	1.91 (1.34-2.74)	<.001^a^
Biopsy	2.09 (1.48-2.95)	<.001^a^	2.10 (1.43-3.07)	<.001^a^
Tumor location				
Hemispheric	1 [Reference]		1 [Reference]	
Midline	1.93 (1.30-2.85)	.001^a^	1.74 (1.16-2.63)	.008^a^
Infratentorial	2.16 (1.47-3.19)	<.001^a^	2.21 (1.48-3.31)	<.001^a^
Tumor histologic grade				
WHO grade III	1 [Reference]		NA	NA
WHO grade IV	1.27 (0.95-1.69)	.11	NA	NA
Adjuvant treatment				
Radiotherapy with chemotherapy	1 [Reference]		1 [Reference]	
Radiotherapy only	2.01 (1.27-3.17)	.003^a^	2.84 (1.67-4.81)	<.001^a^
Chemotherapy only	0.79 (0.47-1.31)	.35	0.77 (0.47-1.28)	.32
None	3.95 (1.61-9.67)	.003^a^	4.93 (2.98-8.16)	<.001^a^

Abbreviations: GTR, gross total resection; HR, hazard ratio; NA, not applicable; STR, subtotal resection; WHO, World Health Organization Classification of Tumors of the Central Nervous System.

^a^ *P* < .05 indicates statistically significant HR of time to mortality.

The outcome of EOR was also examined in location-specific subgroups. Gross total resection was independently associated with longer survival in hemispheric (HR, 0.29; 95% CI, 0.15-0.54; *P* < .001) and infratentorial (HR, 0.44; 95% CI, 0.24-0.83; *P* = .01) tumors compared with STR. However, GTR in patients with midline tumors (HR, 0.63; 95% CI, 0.34-1.19; *P* = .16) was not associated with improved survival (eFigure 7 in the Supplement).

## Discussion

This systematic review and meta-analysis of RCTs and cohort studies of pHGGs found that GTR was independently associated with prolonged survival. Patients with GTR were 45% more likely to survive 1 year and 35% more likely to survive 2 years after tumor resection than patients with STR. Patients with GTR were also 61% more likely to be progression free at 6 months and 35% more likely to be progression free at 1 year after resection. Subtotal resection was associated with survival benefits over biopsy at the 2-year but not at the 1-year follow-up. The number needed to treat was 7.3 for GTR and 4.4 for STR to achieve an additional 1 or 2 years of life. Clinical trial guidelines recommend that an optimal number needed to treat for dichotomous outcomes is lower than 10, suggesting that achieving GTR may extend survival in pHGGs.^63^ Although the association between EOR and survival is well documented in adult HGGs,^6^ no meta-analysis of pHGGs has yet been conducted, to our knowledge. The superiority of GTR over STR and biopsy, lack of superiority between STR and biopsy, and median OS across the 3 EOR subgroups in this study are consistent with results of a large-scale SEER database analysis of 342 pHGGs that was conducted by Adams et al,^19^ whose sample was independent of the IPD cohort in the present study, thus providing quantitative validation of the recommendations for maximal safe resection when technically feasible in pHGGs.^4,24,64,65^ However, this study also estimated that only 29% of patients received GTR, an estimate consistent with those in previous studies (35%-45%).^4,19,66^ The low rate of pursuing GTR in pHGGs is commonly attributed to tumor location because of varying degrees of difficulty across localizations and an incomplete understanding of the association between EOR and tumor survival in different regions, particularly deep-seated infratentorial and midline tumors.^66^ Previous studies have tried to elucidate the latter issue but were limited by underpowered samples.^16,17^ Therefore, we attempted to address this association using a large, pooled sample of IPD.

In the IPDMA, certain subgroups within the study population responded to GTR better than others. Specifically, patients with hemispheric and infratentorial pHGGs experienced prolonged survival after GTR, whereas no survival difference was observed in those with tumors of the midline cerebrum. The classification of tumor location mirrored the post hoc analysis in the clinical trial by the Children’s Cancer Group.^24^ In that trial, Wisoff et al^24^ noted a significant difference in the frequency of radical resection across tumor sites, with the lowest GTR rate and highest biopsy rate occurring in midline tumors, which is consistent with the results of the present study and other studies on thalamic or midline pHGGs.^16,67^ Disparate rates of surgical management in midline pHGGs underscore the difficulty of achieving GTR because of the high risk of postoperative morbidity and mortality from proximity to or infiltration of critical structures.^68^ However, despite the technological advances that have made maximal resection safer and more feasible, there is still no consensus on whether GTR should be pursued in midline tumors.^69,70^ Findings of this study suggest that EOR has no implications for midline pHGGs, but the classification of tumors based on mutations was not captured. Thus, the lack of association between survival and EOR in midline tumors may not be attributed to a lack of efficacy in aggressive surgical management but instead may result from the pooling of clinically and biologically distinct tumors that respond differently to tumor resection.

Thalamic pHGGs are known to represent a distinct clinical subset, as reported by Kramm et al,^67^ and the discovery of *H3K27M*-altered tumors with more aggressive mechanisms of tumorigenesis and substantially worse survival has further stratified this cohort.^11,71,72^ Thus, future investigations of the association between EOR and survival in midline pHGGs should consider the molecular classification of tumors to further elucidate whether and when extensive surgical debulking is worth pursuing in this subgroup. However, given the overwhelming survival benefit associated with GTR in the general population of this study, maximal safe resection—when resection is indicated—should still be pursued for all pHGGs in the meantime.

### Limitations and Strengths

This study has several limitations. First, the lack of differential survival between STR and biopsy may be partly a result of the smaller sample size of the biopsy cohort, suggesting a possible publication bias. Compounding this issue is that STR, for the purposes of this study, included partial tumor resections. Furthermore, we defined EOR according to the judgment of the study authors, which may create bias, imprecision, heterogeneity, and/or overlap between EOR subgroups. However, we attempted to account for these biases by performing a sensitivity analysis that excluded studies with questionable quality, and we found no significant differences in the results.

Second, IPDMAs have inherent limitations because they are still vulnerable to reviewer selection bias, data availability, and publication bias.^73^ The IPD did not capture postoperative functional neurologic status as an outcome because of scarce reporting. The balance between cytoreduction and risk of neurologic morbidity is an important consideration when developing EOR guidelines, especially for children undergoing continued neurodevelopment.^74^ Thus, future studies may evaluate the survival impact of EOR in conjunction with functional status to ensure that protocols and studies encouraging maximal safe resection do not compromise postoperative quality of life.

This study also has some strengths. Extracting IPD in addition to study-level estimates allowed us to perform 2 types of meta-analyses. The results were similar, suggesting reliable representation of the general population and low selection bias. Furthermore, the IPDMA was robust given that the mixed-effects Cox proportional hazards regression model allowed us to control for methodological and clinical variations across studies and covariates associated with survival, including location and adjuvant treatments. Consequently, we were able to mitigate the possibility of aggregation bias commonly observed in study-level meta-analyses.^75^

## Conclusions

To our knowledge, this systematic review and meta-analysis was the first to assess the association between EOR and survival in pHGGs. The results suggest that GTR is independently associated with prolonged OS and PFS compared with STR and biopsy and that STR may not prolong survival compared with biopsy alone. These findings support the pursuit of maximal safe resection when feasible in the treatment of pHGGs. Future studies should seek to identify specific molecular subgroups that benefit most from aggressive cytoreduction.

## Notes

Supplement. **eMethods.** Complete Search Strategy **eTable 1.** Characteristics of Included Studies **eTable 2.** Newcastle-Ottawa Scale and Cochrane Collaboration’s Risk of Bias Tool Evaluation of Articles Included in Meta-analysis **eTable 3.** Sensitivity Analysis of Meta-analysis Results After Removing Subgroups of Studies With Potential Bias **eFigure 1.** PRISMA Flow Diagram **eFigure 2.** Forest Plot of Relative Risk for 1-Year and 2-Year Mortality for Subtotal Resection Versus Biopsy **eFigure 3.** Forest Plot of Relative Risk for 6-Month and 1-Year Progression for Gross Total Resection Versus Subtotal Resection **eFigure 4.** Forest Plot of Relative Risk for 6-Month and 1-Year Progression for Subtotal Resection Versus Biopsy **eFigure 5.** Funnel Plots for Forest Plots of Relative Risk for 1-Year and 2-Year Mortality Between Gross Total Resection Versus Subtotal Resection and Subtotal Resection Versus Biopsy **eFigure 6.** Kaplan-Meier Curve Comparing Overall Survival According to Tumor Location and Adjuvant Treatment Regimen **eFigure 7.** Kaplan-Meier Curve Comparing Overall Survival According to EOR in Tumor Location Subgroups
