# Supplementary material for: Extent of Tumor Resection and Survival in Pediatric Patients With High-Grade Gliomas: A Systematic Review and Meta-analysis
Source: JAMA Netw Open. 2022 Aug 16;5(8):e2226551. doi: 10.1001/jamanetworkopen.2022.26551 (PMC9382445; doi:10.1001/jamanetworkopen.2022.26551)
Supplement: Supplement. — eMethods. Complete Search Strategy eTable 1. Characteristics of Included Studies eTable 2. Newcastle-Ottawa Scale and Cochrane Collaboration’s Risk of Bias Tool Evaluation of Articles Included in Meta-analysis eTable 3. Sensitivity Analysis of Meta-analysis Results After Removing Subgroups of Studies With Potential Bias eFigure 1. PRISMA Flow Diagram eFigure 2. Forest Plot of Relative Risk for 1-Year and 2-Year Mortality for Subtotal Resection Versus Biopsy eFigure 3. Forest Plot of Relative Risk for 6-Month and 1-Year Progression for Gross Total Resection Versus Subtotal Resection eFigure 4. Forest Plot of Relative Risk for 6-Month and 1-Year Progression for Subtotal Resection Versus Biopsy eFigure 5. Funnel Plots for Forest Plots of Relative Risk for 1-Year and 2-Year Mortality Between Gross Total Resection Versus Subtotal Resection and Subtotal Resection Versus Biopsy eFigure 6. Kaplan-Meier Curve Comparing Overall Survival According to Tumor Location and Adjuvant Treatment Regimen eFigure 7. Kaplan-Meier Curve Comparing Overall Survival According to EOR in Tumor Location Subgroups [file jamanetwopen-e2226551-s001.pdf]

Supplemental Online Content

Hatoum R, Chen JS, Lavergne P, et al. Extent of tumor resection and survival in pediatric patients with high-grade gliomas: a systematic review and meta-analysis. *JAMA Netw Open*. 2022;5(8):e2226551. doi:10.1001/jamanetworkopen.2022.26551

eMethods. Complete Search Strategy

eTable 1. Characteristics of Included Studies

eTable 2. Newcastle-Ottawa Scale and Cochrane Collaboration's Risk of Bias Tool Evaluation of Articles Included in Meta-analysis

eTable 3. Sensitivity Analysis of Meta-analysis Results After Removing Subgroups of Studies With Potential Bias

eFigure 1. PRISMA Flow Diagram

eFigure 2. Forest Plot of Relative Risk for 1-Year and 2-Year Mortality for Subtotal Resection Versus Biopsy

eFigure 3. Forest Plot of Relative Risk for 6-Month and 1-Year Progression for Gross Total Resection Versus Subtotal Resection

eFigure 4. Forest Plot of Relative Risk for 6-Month and 1-Year Progression for Subtotal Resection Versus Biopsy

eFigure 5. Funnel Plots for Forest Plots of Relative Risk for 1-Year and 2-Year Mortality Between Gross Total Resection Versus Subtotal Resection and Subtotal Resection Versus Biopsy

eFigure 6. Kaplan-Meier Curve Comparing Overall Survival According to Tumor Location and Adjuvant Treatment Regimen

eFigure 7. Kaplan-Meier Curve Comparing Overall Survival According to EOR in Tumor Location Subgroups

This supplemental material has been provided by the authors to give readers additional information about their work.

© 2022 Hatoum R et al. *JAMA Network Open.*

eMethods. Complete Search Strategy
(#1 AND #2 AND #3) NOT #4

#1: Glioma[Mesh:noexp] OR Oligodendroglioma[mh] OR Ganglioglioma[mh] OR Astrocytoma[Mesh] OR Glioblastoma[tiab] OR glioblastomas[tiab] OR glyoblastoma[tiab] OR glyoblastomas[tiab] OR astroglioma[tiab] OR astrogliomas[tiab] OR astroglyoma[tiab] OR astroglyomas[tiab] OR Astrocytoma[tiab] OR Astrocytomas[tiab] OR oligoastrocytoma[tiab] OR oligoastrocytomas[tiab] OR "glia tumor"[tiab] OR "glia tumors"[tiab] OR "glia tumour"[tiab] OR "glia tumours"[tiab] OR "Glial Cell Tumor"[tiab] OR "Glial Cell Tumors"[tiab] OR "Glial Cell Tumour"[tiab] OR "Glial Cell Tumours"[tiab] OR "glya tumor"[tiab] OR "glya tumors"[tiab] OR "glya tumour"[tiab] OR "glya tumours"[tiab] OR "Glyal Cell Tumor"[tiab] OR "Glyal Cell Tumors"[tiab] OR "Glyal Cell Tumour"[tiab] OR "Glyal Cell Tumours"[tiab] OR Oligodendroglioma[tiab] OR Oligodendrogliomas[tiab] OR glioma[tiab] OR Glyoma[tiab] OR gliomas[tiab] OR Glyomas[tiab] OR Ganglioglioma[tiab] OR Ganglioglyoma[tiab] OR Gangliogliomas[tiab] OR Ganglioglyomas[tiab] OR Glioblastoma[OT] OR glioblastomas[OT] OR glyoblastoma[OT] OR glyoblastomas[OT] OR astroglioma[OT] OR astrogliomas[OT] OR astroglyoma[OT] OR astroglyomas[OT] OR Astrocytoma[OT] OR Astrocytomas[OT] OR oligoastrocytoma[OT] OR oligoastrocytomas[OT] OR "glia tumor"[OT] OR "glia tumors"[OT] OR "glia tumour"[OT] OR "glia tumours"[OT] OR "Glial Cell Tumor"[OT] OR "Glial Cell Tumors"[OT] OR "Glial Cell Tumour"[OT] OR "Glial Cell Tumours"[OT] OR "glya tumor"[OT] OR "glya tumors"[OT] OR "glya tumour"[OT] OR "glya tumours"[OT] OR "Glyal Cell Tumor"[OT] OR "Glyal Cell Tumors"[OT] OR "Glyal Cell Tumour"[OT] OR "Glyal Cell Tumours"[OT] OR Oligodendroglioma[OT] OR Oligodendrogliomas[OT] OR glioma[OT] OR Glyoma[OT] OR gliomas[OT] OR Glyomas[OT] OR Ganglioglioma[OT] OR Ganglioglyoma[OT] OR Gangliogliomas[OT] OR Ganglioglyomas[OT]

#2: Surgery[sh] OR Surgical Oncology[mh] OR General Surgery[mh] OR Neurosurgical Procedures[mh] OR Neoplasm, Residual[mh] OR Resection*[tiab] OR Surger*[tiab] OR Surgical*[tiab] OR Neurosurger*[tiab] OR Neurosurgical*[tiab] OR Radiosurg*[tiab] OR Craniotom*[tiab] OR Craniectom*[tiab] OR Ablation[tiab] OR Remove[tiab] OR Removing[tiab] OR Removal[tiab] OR operation[tiab] OR operative[tiab] OR stereotaxy[tiab] OR Stereotactic[tiab] OR Stereotaxic[tiab] OR residual[tiab] OR Resection*[OT] OR Surger*[OT] OR Surgical*[OT] OR Neurosurger*[OT] OR Neurosurgical*[OT] OR Radiosurg*[OT] OR Craniotom*[OT] OR Craniectom*[OT] OR Ablation[OT] OR Remove[OT] OR Removing[OT] OR Removal[OT] OR operation[OT] OR operative[OT] OR stereotaxy[OT] OR Stereotactic[OT] OR Stereotaxic[OT] OR residual[OT]

#3: Infant[MH] OR Child[MH] OR Adolescent[MH] OR Intensive Care, Neonatal[MH] OR Intensive Care Units, Neonatal[MH] OR Intensive Care Units, Pediatric[MH] OR Hospitals, Pediatric[MH] OR Neonatology[MH] OR Pediatrics[MH] OR Pediatricians[MH] OR Child, Hospitalized[MH] OR Adolescent, Hospitalized[MH] OR newborn*[TIAB] OR new born*[TIAB] OR babie*[TIAB] OR baby*[TIAB] OR infant*[TIAB] OR infancy[TIAB] OR toddler*[TIAB] OR preschool*[TIAB] OR pre school*[TIAB] OR child[TIAB] OR child'[TIAB] OR childs[TIAB] OR child's[TIAB] OR children*[TIAB] OR childhood*[TIAB] OR kid[TIAB] OR kid'[TIAB] OR kids[TIAB] OR kid's[TIAB] OR boy[TIAB] OR boy'[TIAB] OR boys[TIAB] OR boy's[TIAB] OR girl[TIAB] OR girl'[TIAB] OR girls[TIAB] OR girl's[TIAB] OR schoolchild*[TIAB] OR juvenil*[TIAB] OR preadolescen*[TIAB] OR youth*[TIAB] OR adolescen*[TIAB] OR teen[TIAB] OR teen'[TIAB] OR teens[TIAB] OR teen's[TIAB] OR teenage*[TIAB] OR puber[TIAB] OR puber'[TIAB] OR pubers[TIAB] OR puber's[TIAB] OR pubert*[TIAB] OR pubescen*[TIAB] OR high school*[TIAB] OR highschool*[TIAB] OR secondary school*[TIAB] OR paediatric*[TIAB] OR pediatric*[TIAB] OR PICU*[TIAB] OR neonat*[TIAB] OR neo nat*[TIAB] OR NICU*[TIAB] OR newborn*[OT] OR new born*[OT] OR babie*[OT] OR baby*[OT] OR infant*[OT] OR infancy[OT] OR toddler*[OT] OR preschool*[OT] OR pre school*[OT] OR child[OT] OR child'[OT] OR childs[OT] OR child's[OT] OR children*[OT] OR childhood*[OT] OR kid[OT] OR kid'[OT] OR kids[OT] OR kid's[OT] OR boy[OT] OR boy'[OT] OR boys[OT] OR boy's[OT] OR girl[OT]

© 2022 Hatoum R et al. *JAMA Network Open.*

OR girl'[OT] OR girls[OT] OR girl's[OT] OR schoolchild*[OT] OR juvenil*[OT] OR preadolescen*[OT] OR youth*[OT] OR adolescen*[OT] OR teen[OT] OR teen'[OT] OR teens[OT] OR teen's[OT] OR teenage*[OT] OR puber[OT] OR puber'[OT] OR pubers[OT] OR puber's[OT] OR pubert*[OT] OR pubescen*[OT] OR high school*[OT] OR highschool*[OT] OR secondary school*[OT] OR paediatric*[OT] OR pediatric*[OT] OR PICU*[OT] OR neonat*[OT] OR neo nat*[OT] OR NICU*[OT]

#4: Animals[mh] NOT humans[mh]) OR mice[TIAB] OR mices[TIAB] OR mice's[TIAB] OR mices'[TIAB] OR mouse[TIAB] OR mouses[TIAB] OR mouse's[TIAB] OR mouses'[TIAB] OR rat[TIAB] OR rats[TIAB] OR rat's[TIAB] OR rats'[TIAB] OR mice[OT] OR mices[OT] OR mice's[OT] OR mices'[OT] OR mouse[OT] OR mouses[OT] OR mouse's[OT] OR mouses'[OT] OR rat[OT] OR rats[OT] OR rat's[OT] OR rats'[OT]

© 2022 Hatoum R et al. *JAMA Network Open.*

eTable 1. Characteristics of Included Studies

Authors and Year	Study Design	Country	Sample Size	Age	Follow-Up	Extent of Resection Determination	Extent of Resection Definition	Location	Pathology Review	Histology	Adjuvant therapy
Adams et al. 2016 [19]	Retrospective study	USA, UK, Netherlands	336	Mean: 10 years	NS	Medical records	No Surgery (NS), Biopsy (Bx), Partial Resection (PR), Gross Total Resection (GTR), & Surgery Unspecified (SU)	Midline & hemispheric (supratentorial and infratentorial)	NS	Grade IV (100%)	RT (28.6%)<br>CT data not available
Ansari et al. 2012 [30]	Retrospective study	Iran	23	Median: 16 years	NS	Imaging reports	Complete resection (CR), Partial resection (PR), & needle biopsy (NB)	Only hemispheric	NS	Grade IV (100%)	RT (100%)<br>CT (43%)
Bristot et al. 1998 [31]	Retrospective study	Italy	4	Median: 11.5 years	Median: 8.3 months	Post-operative imaging	GTR & STR	Cerebellum	Pathologist diagnostic	Grade III (75%)<br>Grade IV (25%)	RT (50%)<br>CT (0%)
Broniscer et al. 2006 [32]	Non-randomized clinical trial	USA	31	Median: 12.3 years	Median: 15 months	NS	GTR/NTR, STR/PR, & Bx	Hemispheric (42%), thalamic (45%), spinal (9.5%) & posterior fossa (3%)	NS	Grade II (6%)<br>Grade III (44%)<br>Grade IV (48%)	RT (87%)<br>CT (74% TMZ and 64% CPT-11 with TMZ)
Broniscer et al. 2009 [33]	Prospective phase 1 study	USA	23	Median: 10.7 years	NS	NS	GTR, NTR, STR, PR, & Bx	Hemispheric & midline (thalamus, cerebellum)	Central histologic review of all cases conducted by neuropathologist	Grade III (47%)<br>Grade IV (53%)	Local RT<br>CT (Erlotinib administered once daily for up to 3 years)
Chastagner et al. 2007 [34]	Prospective non-randomized study	France	53	Median: 12.1 years	Median: 10.5 years	Pre- and post-operative CT/MRI and neurosurgeon's report	GTR (>90%), PR (25-90%), & Bx (<25%)	Hemispheric & midline (posterior fossa, spinal)	Central pathology review conducted by panel of four neuropathologists	Grade III (30%)<br>Grade IV (38%)<br>Grade III-IV (32%)	RT (100%)<br>CT (BCNU for non-GTR, BCV for GTR)
Di Ruscio et al. 2022 [35]	Retrospective study	Italy	11	Median: 20 months	Median: 24 months	Post-operative MRI	GTR & STR	Hemispheric & midline	Central histologic review conducted by two neuropathologists	Grade III (64%)<br>Grade IV (36%)	RT (18%)<br>CT (100%)
Donson et al. 2007 [36]	Retrospective study	USA	10	Median: 9.9 years	Median: 4.5 months	NS	GTR, STR & Bx	Hemispheric & midline (1 spinal cord tumor)	GBM cell lines established from tumors specimens obtained from surgery	Grade IV (100%)	CT (70% temozolomide)
Dorfer et al. 2021 [37]	Retrospective study	Austria	20	Median: 9.5 years	Median: 1.2 years	Post-operative MRI	STR & Bx	Only thalamic	Central pathology review conducted by neuropathologist	Grade III (65%)<br>Grade IV (35%)	RT (100%)<br>CT (95%)

© 2022 Hatoum R et al. *JAMA Network Open.*

Dufour et al. 2006 [38]	Prospective study	France	21	Median: 23 months	Median: 5.2 years	Early imaging-documented extent of resection	GTR (absence of residual tumor), STR (>90%), PR (<90%), & Bx	Hemispheric & midline (and posterior fossa)	Centrally reviewed by panel of neuropathologists, WHO grading	Grade III (62%)<br>Grade IV (38%)	RT (0%)<br>CT (100%)
Eisenstat et al. 2014 [39]	Retrospective study	USA, Canada	60	Mean: 7.61 years	NS	Surgical reports (centrally reviewed)	GTR/NTR (>90% resection), STR (50-90%), PR (10-50%), & Bx (<10%)	Midline (basal ganglia, hypothalamus, thalamus, brainstem, other)	Central pathology review conducted by panel of five neuropathologist	Grade III & IV	RT (100%)<br>CT (either eight cycles of CCNU, vincristine and prednisone or vincristine, hydroxyurea, procarbazine, cisplatin, cytarabinoside, dacarbazine and methylprednisolone)
Fernandez et al. 2006 [40]	Retrospective study	France	14	Median: 8 years	Median: 12 months	Surgical reports and post-operative imaging	GTR, PR, & Bx	Only thalamic	Centrally reviewed, WHO grading	Grade II (43%)<br>Grade III (43%)<br>Grade IV (14%)	RT (42%)<br>CT (57%),
Geyer et al. 1995 [41]	Prospective non-randomized trial	USA	32	Mean: 10 months	NS	Post-operative CT scan and operating neurosurgeon's classification	Total resection (>90%), STR (59-90%), PR (<50%) & Bx	Hemispheric & midline (spinal and posterior fossa)	Centrally reviewed	Grade III & IV	RT (7%)<br>CT (100%)
Grovas et al. 1999 [42]	Prospective phase II study	USA	11	Median: 12 years	NS	NS	Complete resection (>90%), STR (51-90%)	NS	Histologic diagnosis was confirmed by rapid review process before study entry	Grade IV (100%)	Delayed RT (100%) The pre-ASCR preparative regimen included BCNU; thiotepa and etoposide.
Heideman et al. 1993 [43]	Non-randomized prospective study	USA	13	Median: 10 years	Median: 14 months	NS	NTR (>90%), STR (50-90%), & Bx	NS	NS	Grade III (38%)<br>Grade IV (62%)	RT (84%)<br>CT (100% thiotepa, cyclophosphamide and autologous bone marrow infiltration)
Jakacki et al. 1999 [44]	Non-randomized prospective study	USA	12	Median: 7.5 years	Median: 8.5 months	NS	GTR, STR, PR, & Bx	Hemispheric & midline (1 spinal cord)	NS	Grade III (17%)<br>Grade IV (33%)<br>Non-specified (50%)	RT (100%)<br>CT (100% PCV)
Jakacki et al. 2008 [45]	Non-randomized prospective study	USA	32	Median: 12.5 years	Median: 15 months	NS	GTR, NTR, STR, PR, & Bx	Hemispheric & midline	Central pathology review	Grade III (34%)<br>Grade IV (50%)	RT (88%)<br>CT (88% temozolomide & lomustine)

© 2022 Hatoum R et al. *JAMA Network Open.*

Jiao et al. 2021 [46]	Retrospective study	China	38	Median: 11.5 years	Mean: 13 months	NS	GTR & STR	Hemispheric & midline	NS	Grade IV (100%)	RT (2.6%)<br>CT (13.2%)<br>RT + CT (47.4%)
Jung et al. 2015 [47]	Retrospective multicenter study	South Korea	62	Median: 12 years	NS	Post-operative CT or MRI scan	GTR (no residual tumor), STR (<50% residual tumor), PR (>50% residual tumor), & Bx	Hemispheric & midline	Grading according to WHO classification	Grade III (45%)<br>Grade IV (55%)	RT 16.1%)<br>CT (1.6%)<br>RT + CT (53.3%)
Kramm et al. 2006 [48]	Retrospective study	USA, Canada, Germany, Suisse, Austria, and Belgium	85	Median: 11 years	Median: 12 months	Neurosurgeon's reports and post-operative MRI	GTR (100% macroscopic tumor resection), STR (90%-100%), PR (<90%), & Bx	Hemispheric (cortical) & midline (cerebellar and other)	Central pathology review	Grade III (40%)<br>Grade IV (60%)	RT (94%)<br>CT (94%)
Kulkarni et al. 1999 [49]	Retrospective study	USA	4	Median: 5.4 years	Mean: 13 months	NS	GTR & STR	Cerebellum	NS	Grade IV (100%)	RT (50%)<br>CT(50%)
Mahvash et al. 2011 [50]	Retrospective study	Germany	13	Mean: 10.4 years	NS	Cranial CT scan (1991-2002) within 24 hours after surgery or MRI (2002-2005) within 72 hours after surgery	GTR & STR	Hemispheric & midline (cerebellum, brainstem)	Diagnosis was verified by neuropathologist	Grade IV (100%)	RT (85%)<br>CT (70%)
Masoudi et al. 2008 [51]	Retrospective study	USA	66	Median: 12.5 years	Median: 1 year	NS	GTR, STR, PR, & Bx	NS	NS	Grade III (39%)<br>Grade IV (61%)	RT + CT
McCrea et al. 2015 [16]	Retrospective study	USA	97	Median: 11 years	Mean: 2.3 years	Postoperative imaging (magnetic resonance imaging [MRI]) report or from the clinical record.	GTR, STR, & Bx	Hemispheric (supratentorial) & midline (cerebellum, thalamus, brainstem)	Pathologist diagnostic	Grade III & IV	Excluding 13 patients with unknown adjuvant therapy: 92% of patients in our study were treated with radiation, 93% with chemotherapy, and 88% with both chemotherapy and radiation.

© 2022 Hatoum R et al. *JAMA Network Open.*

Nikitovic et al. 2015 [18]	Retrospective study	Serbia	15	Mean: 11.8 years	NS	Surgeon's operative report and postoperative CT/MRI images	GTR & STR	Hemispheric & midline (infratentorial, supratentorial)	Neuropathologist reviewed all slides and confirmed histopathological diagnosis	Grade IV (100%)	RT (100%)<br>CT (60%)
Papadakis et al. 1999 [52]	Non-randomized prospective study	USA	42	Median: 12.2 years	Median: 11 months	Imaging reports	Radical resection, less than radical resection, & Bx	Hemispheric & midline (2 spinal cord tumors)	NS	Grade III (30%)<br>Grade IV (70%)	RT (50% post bone marrow rescue)<br>CT (100% thiotepa, BCNU & VP-16 prior to bone marrow rescue)
Parekh et al. 2010 [53]	Retrospective study	USA	8	Median: 13.5 years	Median: 33 weeks	NS	GTR, NTR, STR, & PR	Hemispheric & midline (thalamus, spine, brainstem)	NS	Grade III (75%)<br>Grade IV (12%)	Six patients had received radiotherapy and all patients had received chemotherapy prior to the recurrence / progression for which a bevacizumab containing regimen was administered
Perkins et al. 2011 [54]	Retrospective study	USA	24	Median: 11 years	Median: 13 months	Qualitatively by surgeon and MRI when possible	GTR, STR, & Bx	Hemispheric & midline (infratentorial, supratentorial)	NS	Grade IV (100%)	RT (100%)<br>CT (58%)
Phuphanich et al. 1984 [55]	Retrospective study	USA	27	Median: 12.1 years	Median: 2.5 years	NS	STR & Bx	Hemispheric & midline	Histological diagnosis confirmed at surgery	Grade III & IV	RT (100%)<br>CT (44%)
Reddy et al. 2012 [56]	Retrospective study	USA	5	Median: 5 years	Mean: 16 months	Surgery report	GTR & STR	Cerebellum	Histological diagnosis	Grade IV (100%)	RT (80%)<br>CT (60%),
Rodriguez et al. 2022 [57]	Post-hoc analysis of a prospective trial	Multicenter	43	Median: 12.1 years	Median: 1 year	Post-operative MRI	Total, major debulking, minor debulking, & Bx	Only thalamic	Central histology review by one neuropathologist	Grade III (37.2%)<br>Grade IV (62.8%)	RT (100%)<br>CT (100%)
Sanders et al. 2007 [58]	Retrospective study	USA	16	Median: 1.1 years	Median: 11.6 years	NS	GTR, NTR, STR. & Bx	Hemispheric & midline	Pathology reviewed by two independent pathologists	Grade III (56%)<br>Grade IV (31%)	RT (80%)<br>CT (100%)
Shinoda et al. 1989 [59]	Retrospective study	Japan	5	Mean: 11 years	NS	NS	STR (70-90%) & Bx	Cerebellum	NS	Grade III (40%)<br>Grade IV (60%)	RT (80%)<br>CT (80% bleomycin, adriamycin, ACNU, vincristine),

© 2022 Hatoum R et al. *JAMA Network Open.*

Sirachainan et al. 2017 [60]	Prospective cohort study	Thailand	16	Mean: 8.2 years	Median: 1 year	NS	GTR, NTR, PR, & Bx	Hemispheric & midline	NS	Grade III (37%)<br>Grade IV (63%)	RT (100%)<br>CT (100%, Nimotuzumab and irinotecan)
Sposto et al. 1989 [61]	Randomized controlled trial	USA	58	NS	NS	CT scans were not universally available, Surgical checklists and surgical notes were reviewed centrally	Complete resection, PR, & Bx	Hemispheric & midline (posterior fossa, basal ganglia)	Central pathology review performed on all patient	Grade III (46%)<br>Grade IV (54%)	RT + CT (48%)<br>RT alone (51%)
Wu et al. 2016 [62]	Retrospective study	Taiwan	20	Median: 9.2 years	Median: 5.3 years	Operative records of the surgeons and neuroimaging data 3 days after operation	GTR, STR, & Bx	Hemispheric & midline (spine, suprasellar, intraventricular)	Pathologist diagnostic	Grade II (45%)<br>Grade III (55%)	RT (35%)<br>CT (5%)
Yang et al. 2013 [15]	Retrospective study	USA	37	Median: 10.8 years	NS	MRI when available, otherwise surgeon estimations or CT	GTR (100%), STR (<99%), & Bx	Hemispheric & midline (defined as cortical and deep). Excluded brainstem	NS	Grade IV (100%)	RT only (5.4%)<br>CT only (2.7%)<br>RT + CT (89.2%)

© 2022 Hatoum R et al. *JAMA Network Open.*

eTable 2. Newcastle-Ottawa Scale and Cochrane Collaboration’s Risk of Bias Tool Evaluation of Articles Included in Meta-analysis

	Newcastle-Ottawa Scale								
	Selection				Comparability	Outcome			
Author & Year.	Representativeness of exposed cohort	Selection of non-exposed cohort	Ascertainment of exposure	Demonstration that outcome of interest was not present at start of study	Comparability of cohorts on the basis of the design or analysis	Assessment of outcome	Follow-up long enough for outcome to occur	Adequacy of follow-up	Total
Adams et al. 2016 [19]		*	*		**	*	*	*	7
Ansari et al. 2012 [30]	*	*	*		*	*	*		6
Bristot et al. 1998 [31]		*	*			*		*	4
Broniscer et al. 2006 [32]		*				*	*	*	4
Broniscer et al. 2009 [33]		*				*			2
Chastagner et al. 2006 [34]	*	*	*			*	*		5
Di Ruscio et al. 2022 [35]		*	*		*	*	*		5
Donson et al. 2007 [36]	*	*			*	*	*	*	6
Dorfer et al. 2021 [37]		*	*		*	*	*		5
Dufour et al. 2006 [38]		*	*			*	*	*	5
Eisenstat et al. 2014 [39]		*	*			*	*		4
Fernandez et al. 2006 [40]		*	*			*	*	*	5
Geyer et al. 1995 [41]		*	*			*	*		4
Grovas et al. 1999 [42]		*				*	*		3
Heideman et al. 1993 [43]		*				*	*		3
Jakacki et al. 1999 [44]		*				*	*	*	4
Jakacki et al. 2008 [45]		*				*	*	*	4
Jiao et al. 2021 [46]	*	*			*	*	*		5
Jung et al. 2015 [47]	*	*	*		**	*	*		7
Kramm et al. 2006 [48]	*	*	*		**	*	*		7
Kulkarni et al. 1999 [49]	*	*				*	*		4
Mahvash et al. 2011 [50]	*	*	*			*	*		5
Masoudi et al. 2008 [51]	*	*				*	*		4
McCrea et al. 2015 [16]	*	*	*		*	*	*		6
Nikitovic et al. 2015 [18]	*	*	*			*	*	*	6
Papadakis et al. 1999 [52]		*	*			*	*	*	5
Parekh et al. 2010 [53]		*				*			2
Perkins et al. 2011 [54]	*	*	*			*	*	*	6
Phuphanich et al. 1984 [55]	*	*			*	*	*		5
Reddy et al. 2012 [56]		*	*			*	*		4
Rodriguez et al. 2022 [57]		*	*		*	*	*	*	6
Sanders et al. 2007 [58]	*	*			*	*	*	*	6
Shinoda et al. 1989 [59]		*				*	*	*	4
Sirachainan et al. 2017 [60]	*	*	*			*	*		5
Wu et al. 2016 [62]	*	*	*			*	*	*	6
Yang et al. 2013 [15]	*	*	*			*	*	*	6
	Cochrane Collaboration’s Risk Bias Tool								
	Random sequence generation	Allocation concealment	Blinding of participant and personnel	Blinding of outcome assessment	Incomplete outcome data	Selective reporting	Other bias		
Sposto et al. 1989 [61]	unclear	unclear	high	high	low	low	low		

© 2022 Hatoum R et al. *JAMA Network Open.*

eTable 3. Sensitivity Analysis of Meta-analysis Results After Removing Subgroups of Studies With Potential Bias

Comparison	RR overall	RR without SEER data	RR without studies that have a quality grade < 6	RR without data manually extracted from Kaplan-Meier
1-year RR mortality GTR vs STR	0.69 [0.56-0.83] *p*<0.001	0.65 [0.51-0.83] *p*<0.001	0.66 [0.51-0.85] *p*=0.001	0.73 [0.59-0.91] *p*=0.006
2-year RR mortality GTR vs STR	0.74 [0.67-0.83] *p*<0.001	0.74 [0.64-0.87] *p*<0.001	0.69 [0.60-0.81] *p*<0.001	0.76 [0.67-0.87] *p*<0.001
1-year RR mortality STR vs Bx	0.82 [0.66-1.01] *p*=0.07	0.80 [0.63-1.03] *p*=0.08	0.80 [0.60-1.06] *p*=0.12	0.94 [0.76-1.17] *p*=0.57
2-year RR mortality STR vs Bx	0.89 [0.82-0.97] *p*=0.01	0.85 [0.78-0.94] *p*=0.001	0.86 [0.75-1.00] *p*=0.05	0.99 [0.89-1.10] *p*=0.80

© 2022 Hatoum R et al. *JAMA Network Open.*

eFigure 1. PRISMA Flow Diagram

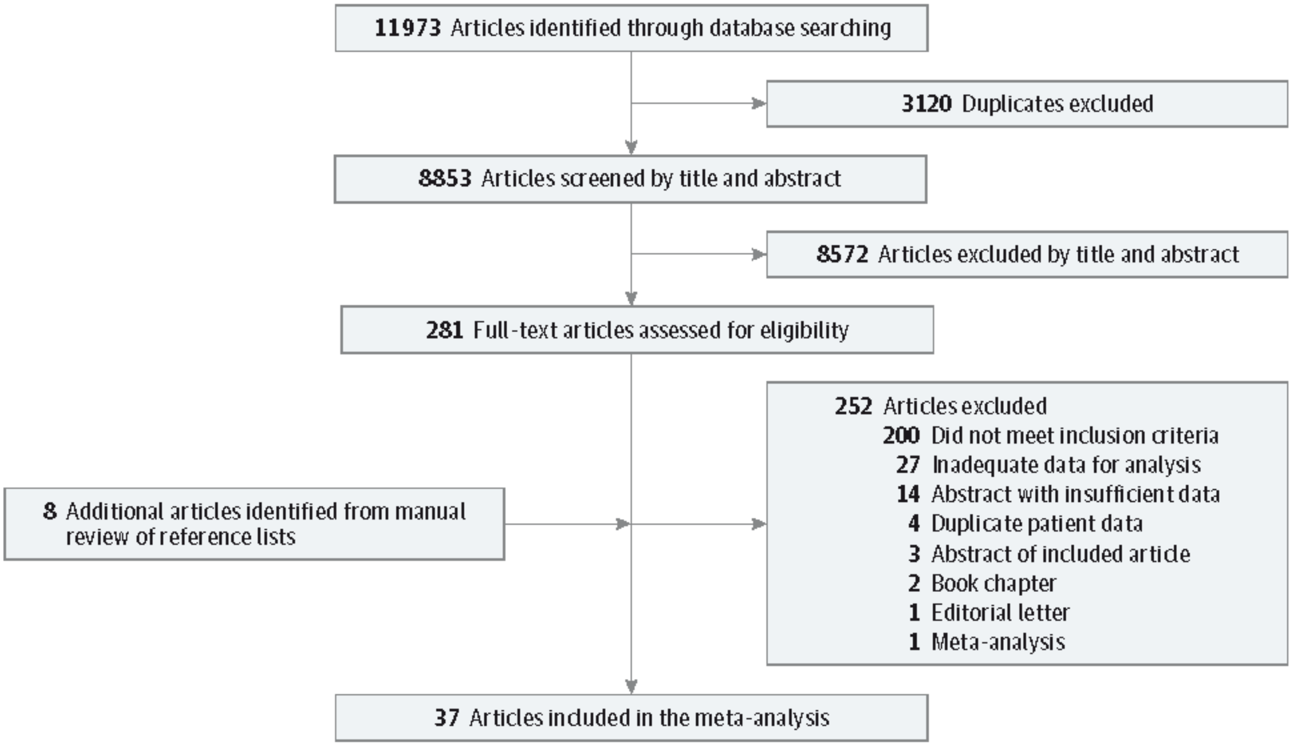

© 2022 Hatoum R et al. *JAMA Network Open.*

eFigure 2. Forest Plot of Relative Risk for 1-Year and 2-Year Mortality for Subtotal Resection Versus Biopsy

A.) 1-Year Mortality

Study	STR Events	STR Total	Biopsy Events	Biopsy Total	Weight	Risk Ratio MH, Random, 95% CI
Adams et al, 2016 [19]	47	97	30	58	17.0%	0.94 [0.68; 1.29]
Ansari et al, 2012 [30]	5	9	7	12	6.3%	0.95 [0.45; 2.03]
Broniscer et al, 2006 [32]	3	10	4	9	2.9%	0.68 [0.20; 2.23]
Donson et al, 2007 [36]	0	1	1	2	0.6%	0.50 [0.04; 7.10]
Dorfer et al, 2021 [37]	4	12	4	8	3.6%	0.67 [0.23; 1.92]
Dufour et al, 2006 [38]	3	11	1	3	1.3%	0.82 [0.13; 5.30]
Eisenstat et al, 2014 [39]	12	29	6	26	5.5%	1.79 [0.79; 4.09]
Fernandez et al, 2006 [40]	4	5	1	2	2.1%	1.60 [0.37; 6.85]
Geyer et al, 1995 [41]	6	13	1	3	1.5%	1.38 [0.25; 7.61]
Heideman et al, 1993 [43]	2	4	2	6	1.9%	1.50 [0.34; 6.70]
Jakacki et al, 2008 [45]	3	9	3	9	2.5%	1.00 [0.27; 3.69]
Kramm et al, 2006 [48]	14	37	14	23	10.4%	0.62 [0.37; 1.05]
Masoudi et al, 2008 [51]	5	39	7	14	4.2%	0.26 [0.10; 0.68]
McCrea et al, 2015 [16]	13	42	6	23	5.5%	1.19 [0.52; 2.70]
Papadakis et al, 1999 [52]	3	8	3	4	3.6%	0.50 [0.17; 1.44]
Perkins et al, 2011 [54]	5	12	2	5	2.6%	1.04 [0.29; 3.69]
Phuphanich et al, 1984 [55]	3	13	2	13	1.7%	1.50 [0.30; 7.55]
Rodriguez et al, 2022 [57]	6	11	9	18	6.9%	1.09 [0.54; 2.22]
Sanders et al, 2007 [58]	1	2	0	3	0.6%	4.00 [0.24; 67.71]
Shinoda et al, 1989 [59]	3	4	1	1	4.1%	0.93 [0.35; 2.50]
Sposto et al, 1989 [61]	11	39	9	11	9.4%	0.34 [0.19; 0.61]
Wu et al, 2016 [62]	1	3	0	2	0.6%	2.25 [0.13; 38.09]
Yang et al, 2013 [15]	6	13	4	7	5.0%	0.81 [0.34; 1.93]
**Total (95% CI)**		**423**		**262**	**100.0%**	**0.82 [0.66; 1.01]**

Heterogeneity: $Tau^2 = 0.0446$; $Chi^2 = 26.84$, df = 22 (P = 0.22); $I^2 = 18\%$
Test for overall effect: Z = -1.83 (P = 0.07)

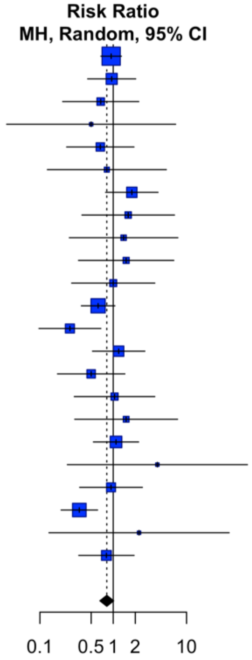

B.) 2-Year Mortality

Study	STR Events	STR Total	Biopsy Events	Biopsy Total	Weight	Risk Ratio MH, Random, 95% CI
Adams et al, 2016 [19]	77	97	42	58	17.1%	1.10 [0.91; 1.32]
Ansari et al, 2012 [30]	8	9	11	12	8.4%	0.97 [0.73; 1.29]
Broniscer et al, 2006 [32]	9	10	8	9	7.3%	1.01 [0.74; 1.38]
Donson et al, 2007 [36]	1	1	2	2	0.9%	0.90 [0.35; 2.32]
Dorfer et al, 2021 [37]	8	12	8	8	4.0%	0.69 [0.45; 1.06]
Dufour et al, 2006 [38]	5	11	1	3	0.3%	1.36 [0.24; 7.66]
Eisenstat et al, 2014 [39]	18	29	19	26	5.3%	0.85 [0.59; 1.23]
Fernandez et al, 2006 [40]	5	5	2	2	2.4%	1.10 [0.63; 1.93]
Geyer et al, 1995 [41]	7	13	1	3	0.3%	1.62 [0.30; 8.65]
Heideman et al, 1993 [43]	3	3	4	5	2.1%	1.17 [0.65; 2.11]
Jakacki et al, 2008 [45]	7	9	6	9	2.2%	1.17 [0.65; 2.08]
Kramm et al, 2006 [48]	27	37	20	23	10.6%	0.84 [0.65; 1.08]
Masoudi et al, 2008 [51]	25	39	12	14	7.0%	0.75 [0.54; 1.03]
McCrea et al, 2015 [16]	25	42	19	23	7.2%	0.72 [0.53; 0.98]
Papadakis et al, 1999 [52]	4	8	3	4	1.0%	0.67 [0.27; 1.63]
Perkins et al, 2011 [54]	8	11	4	5	2.3%	0.91 [0.51; 1.60]
Phuphanich et al, 1984 [55]	4	12	4	12	0.6%	1.00 [0.32; 3.10]
Rodriguez et al, 2022 [57]	9	10	16	16	10.8%	0.89 [0.69; 1.14]
Sanders et al, 2007 [58]	1	2	0	3	0.1%	4.00 [0.24; 67.71]
Shinoda et al, 1989 [59]	4	4	1	1	1.1%	1.20 [0.51; 2.81]
Sposto et al, 1989 [61]	18	39	10	11	4.8%	0.51 [0.34; 0.75]
Wu et al, 2016 [62]	1	3	1	2	0.2%	0.67 [0.08; 5.54]
Yang et al, 2013 [15]	10	13	6	7	4.1%	0.90 [0.59; 1.37]
**Total (95% CI)**		**419**		**258**	**100.0%**	**0.89 [0.82; 0.97]**

Heterogeneity: $Tau^2 = 0.0025$; $Chi^2 = 23.29$, df = 22 (P = 0.39); $I^2 = 6\%$
Test for overall effect: Z = -2.57 (P = 0.01)

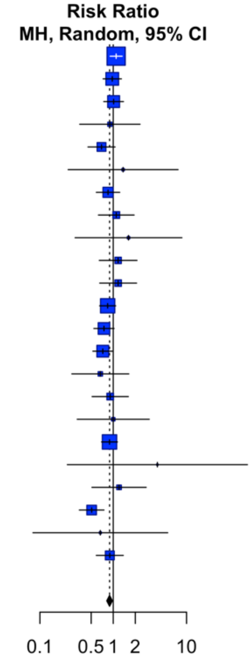

© 2022 Hatoum R et al. *JAMA Network Open.*

eFigure 3. Forest Plot of Relative Risk for 6-Month and 1-Year Progression for Gross Total Resection Versus Subtotal Resection

A.) 6-Month Progression

Study	GTR Events	GTR Total	STR Events	STR Total	Weight	Risk Ratio MH, Random, 95% CI
Broniscer et al, 2009 [33]	1	11	2	8	1.7%	0.36 [0.04; 3.35]
Chastagner et al, 2007 [34]	0	8	10	30	1.1%	0.16 [0.01; 2.54]
Di Ruscio et al, 2022 [35]	0	4	2	3	1.1%	0.16 [0.01; 2.47]
Donson et al, 2007 [36]	3	5	1	1	7.5%	0.78 [0.27; 2.22]
Dufour et al, 2006 [38]	1	3	3	9	2.4%	1.00 [0.16; 6.35]
Eisenstat et al, 2014 [39]	1	5	9	29	2.4%	0.64 [0.10; 4.04]
Geyer et al, 1995 [41]	5	12	8	13	13.0%	0.68 [0.31; 1.50]
Heideman et al, 1993 [43]	0	2	0	4	0.0%	
Jakacki et al, 1999 [44]	1	2	2	2	5.3%	0.60 [0.17; 2.07]
Jiao et al, 2021 [46]	6	16	15	22	17.1%	0.55 [0.27; 1.10]
Jung et al, 2015 [47]	4	20	2	13	3.4%	1.30 [0.28; 6.11]
Kramm et al, 2006 [48]	5	25	13	37	10.2%	0.57 [0.23; 1.40]
McCrea et al, 2015 [16]	9	32	18	42	19.2%	0.66 [0.34; 1.26]
Papadakis et al, 1999 [52]	0	4	1	6	0.9%	0.47 [0.02; 9.26]
Parekh et al, 2010 [53]	0	4	1	2	1.0%	0.20 [0.01; 3.50]
Rodriguez et al, 2022 [57]	4	10	8	13	10.8%	0.65 [0.27; 1.56]
Sanders et al, 2007 [58]	3	11	1	2	2.9%	0.55 [0.10; 2.95]
**Total (95% CI)**		**174**		**236**	**100.0%**	**0.62 [0.46; 0.82]**

Heterogeneity: $Tau^2 = 0$; $Chi^2 = 4.41$, df = 15 (P = 1.00); $I^2 = 0\%$
Test for overall effect: Z = -3.30 (P < 0.01)

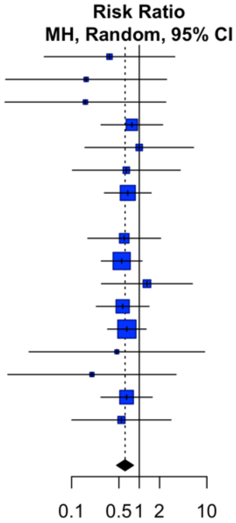

B. 1-Year Progression

Study	GTR Events	GTR Total	STR Events	STR Total	Weight	Risk Ratio MH, Random, 95% CI
Broniscer et al, 2009 [33]	4	11	5	8	4.0%	0.58 [0.23; 1.50]
Chastagner et al, 2007 [34]	1	8	21	30	1.2%	0.18 [0.03; 1.13]
Di Ruscio et al, 2022 [35]	0	4	3	3	0.6%	0.11 [0.01; 1.63]
Donson et al, 2007 [36]	5	5	1	1	5.0%	1.22 [0.53; 2.82]
Dufour et al, 2006 [38]	3	3	6	9	8.8%	1.35 [0.75; 2.42]
Eisenstat et al, 2014 [39]	2	5	15	29	2.9%	0.77 [0.25; 2.39]
Geyer et al, 1995 [41]	5	12	7	13	5.0%	0.77 [0.33; 1.79]
Heideman et al, 1993 [43]	0	2	4	4	0.6%	0.19 [0.01; 2.36]
Jakacki et al, 1999 [44]	2	2	2	2	6.4%	1.00 [0.49; 2.05]
Jiao et al, 2021 [46]	11	16	21	22	17.1%	0.72 [0.51; 1.01]
Jung et al, 2015 [47]	8	20	13	13	10.2%	0.42 [0.25; 0.71]
Kramm et al, 2006 [48]	11	25	28	37	11.6%	0.58 [0.36; 0.94]
McCrea et al, 2015 [16]	16	32	25	42	13.4%	0.84 [0.55; 1.29]
Papadakis et al, 1999 [52]	2	4	3	6	2.4%	1.00 [0.28; 3.54]
Parekh et al, 2010 [53]	1	4	1	2	0.8%	0.50 [0.06; 4.47]
Rodriguez et al, 2022 [57]	6	10	9	12	8.4%	0.80 [0.44; 1.46]
Sanders et al, 2007 [58]	5	11	1	2	1.7%	0.91 [0.20; 4.20]
**Total (95% CI)**		**174**		**235**	**100.0%**	**0.74 [0.60; 0.90]**

Heterogeneity: $Tau^2 = 0.0318$; $Chi^2 = 19.87$, df = 16 (P = 0.23); $I^2 = 19\%$
Test for overall effect: Z = -2.96 (P < 0.01)

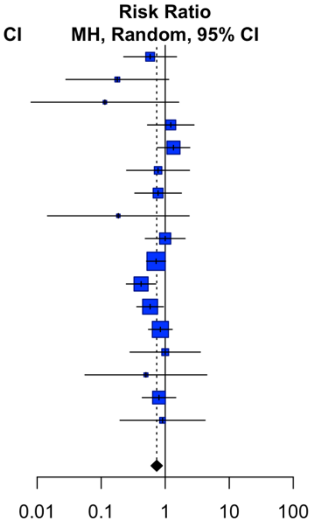

© 2022 Hatoum R et al. *JAMA Network Open.*

eFigure 4. Forest Plot of Relative Risk for 6-Month and 1-Year Progression for Subtotal Resection Versus Biopsy

A.) 6-Month Progression

Study	STR Events	STR Total	Biopsy Events	Biopsy Total	Weight	Risk Ratio MH, Random, 95% CI
Broniscer et al, 2009 [33]	2	8	1	4	2.4%	1.00 [0.13; 8.00]
Chastagner et al, 2007 [34]	10	30	7	15	11.2%	0.71 [0.34; 1.50]
Donson et al, 2007 [36]	2	2	1	2	5.7%	1.67 [0.48; 5.76]
Dufour et al, 2006 [38]	3	9	2	2	7.9%	0.42 [0.16; 1.12]
Eisenstat et al, 2014 [39]	9	29	6	26	9.1%	1.34 [0.55; 3.27]
Geyer et al, 1995 [41]	5	13	2	3	7.2%	0.58 [0.20; 1.66]
Heideman et al, 1993 [43]	0	4	2	6	1.4%	0.28 [0.02; 4.66]
Jakacki et al, 1999 [44]	2	2	1	1	8.3%	1.11 [0.43; 2.86]
Jung et al, 2015 [47]	2	13	0	29	1.2%	10.71 [0.55; 208.71]
Kramm et al, 2006 [48]	13	37	14	23	14.8%	0.58 [0.33; 1.00]
McCrea et al, 2015 [16]	18	42	9	23	13.4%	1.10 [0.59; 2.03]
Papadakis et al, 1999 [52]	1	6	3	3	4.3%	0.24 [0.06; 1.06]
Phuphanich et al, 1984 [55]	3	14	1	13	2.3%	2.79 [0.33; 23.52]
Rodriguez et al, 2022 [57]	8	13	5	16	9.6%	1.97 [0.85; 4.58]
Sanders et al, 2007 [58]	1	2	0	3	1.3%	4.00 [0.24; 67.71]
**Total (95% CI)**		**224**		**169**	**100.0%**	**0.91 [0.65; 1.28]**

Heterogeneity: $Tau^2 = 0.1220$; $Chi^2 = 20.36$, df = 14 (P = 0.12); $I^2 = 31\%$

Test for overall effect: Z = -0.54 (P = 0.59)

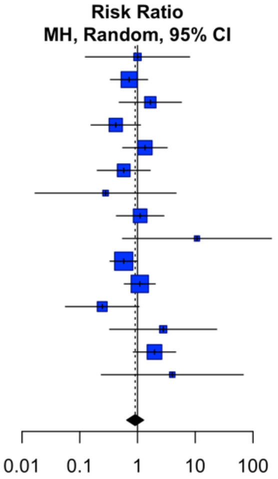

B.) 1-Year Progression

Study	STR Events	STR Total	Biopsy Events	Biopsy Total	Weight	Risk Ratio MH, Random, 95% CI
Broniscer et al, 2009 [33]	5	8	2	4	1.3%	1.25 [0.41; 3.82]
Chastagner et al, 2007 [34]	21	30	10	15	8.3%	1.05 [0.68; 1.61]
Donson et al, 2007 [36]	1	1	2	2	1.8%	0.90 [0.35; 2.32]
Dufour et al, 2006 [38]	6	9	2	2	3.4%	0.78 [0.40; 1.54]
Eisenstat et al, 2014 [39]	15	29	14	26	6.1%	0.96 [0.58; 1.58]
Geyer et al, 1995 [41]	7	13	2	3	1.8%	0.81 [0.31; 2.08]
Heideman et al, 1993 [43]	4	4	3	6	2.5%	1.80 [0.81; 3.99]
Jakacki et al, 1999 [44]	2	2	1	1	1.8%	1.11 [0.43; 2.86]
Jung et al, 2015 [47]	13	13	21	29	23.1%	1.35 [1.05; 1.72]
Kramm et al, 2006 [48]	28	37	20	23	23.9%	0.87 [0.68; 1.11]
McCrea et al, 2015 [16]	25	42	15	23	10.0%	0.91 [0.62; 1.35]
Papadakis et al, 1999 [52]	3	6	3	3	2.3%	0.57 [0.25; 1.31]
Phuphanich et al, 1984 [55]	5	13	5	13	1.7%	1.00 [0.38; 2.64]
Rodriguez et al, 2022 [57]	9	12	15	16	12.1%	0.80 [0.56; 1.14]
Sanders et al, 2007 [58]	1	2	0	3	0.2%	4.00 [0.24; 67.71]
**Total (95% CI)**		**221**		**169**	**100.0%**	**1.00 [0.88; 1.13]**

Heterogeneity: $Tau^2 = 0.0020$; $Chi^2 = 14.45$, df = 14 (P = 0.42); $I^2 = 3\%$

Test for overall effect: Z = -0.06 (P = 0.95)

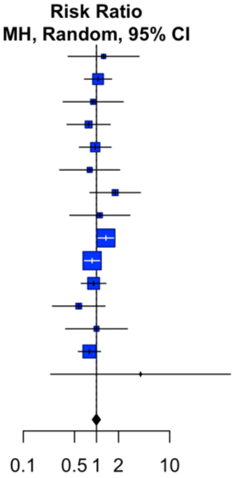

© 2022 Hatoum R et al. *JAMA Network Open.*

eFigure 5. Funnel Plots for Forest Plots of Relative Risk for 1-Year and 2-Year Mortality Between Gross Total Resection Versus Subtotal Resection and Subtotal Resection Versus Biopsy

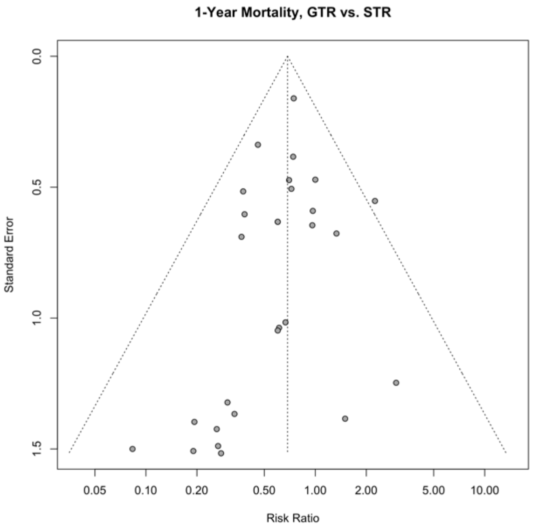

A.)

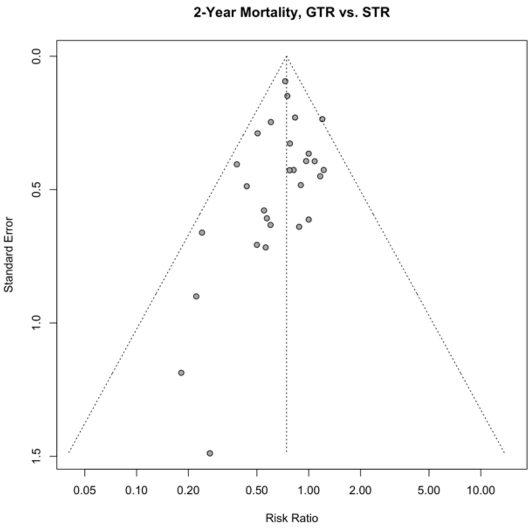

B.)

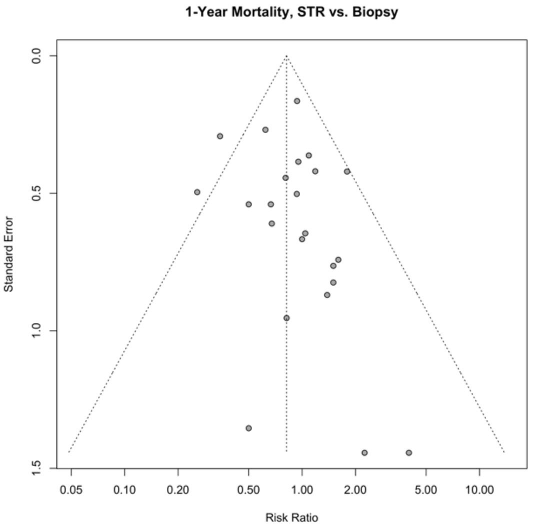

C.)

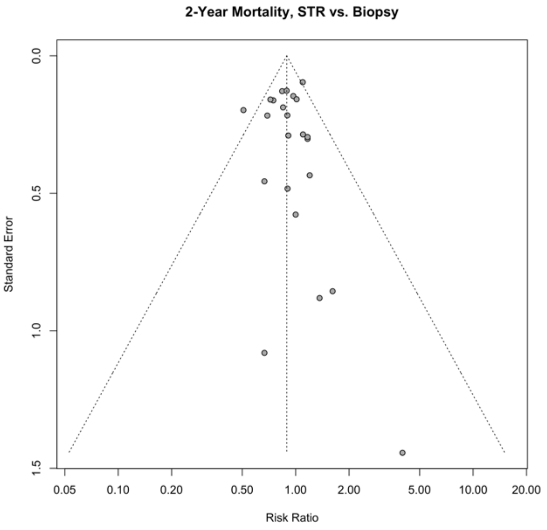

D.)

© 2022 Hatoum R et al. *JAMA Network Open.*

eFigure 6. Kaplan-Meier Curve Comparing Overall Survival According to Tumor Location and Adjuvant Treatment Regimen

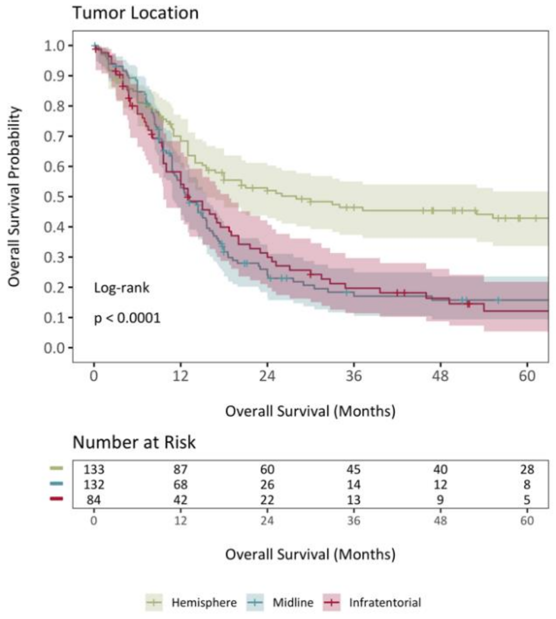

A.)

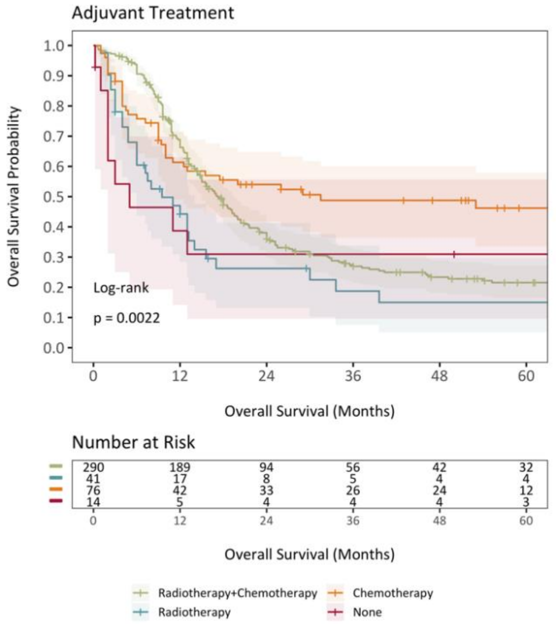

B.)

© 2022 Hatoum R et al. *JAMA Network Open.*

eFigure 7. Kaplan-Meier Curve Comparing Overall Survival According to EOR in Tumor Location Subgroups

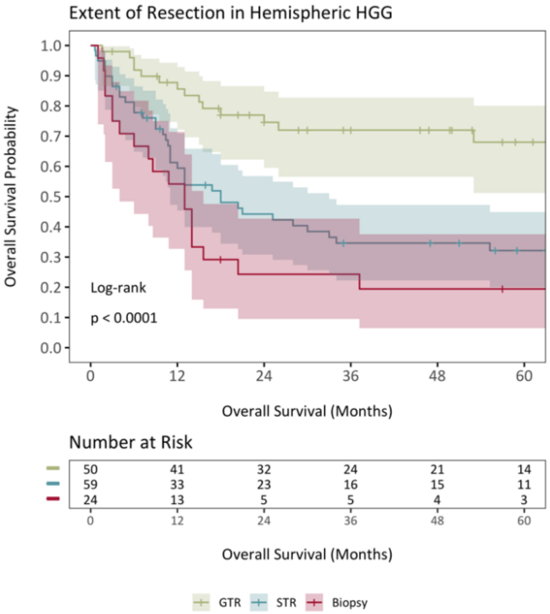

A.)

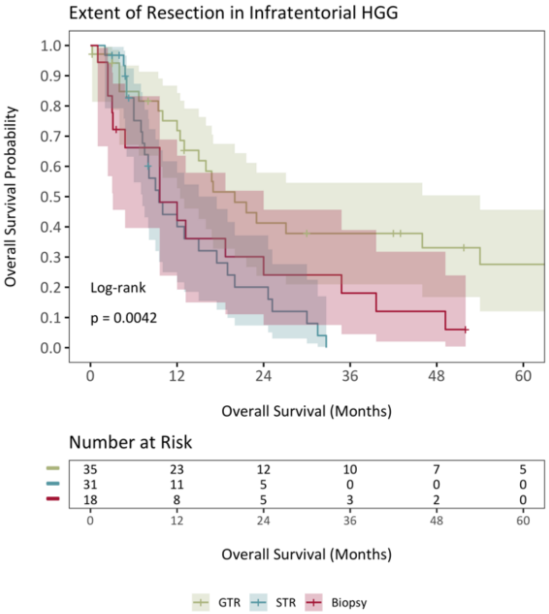

B.)

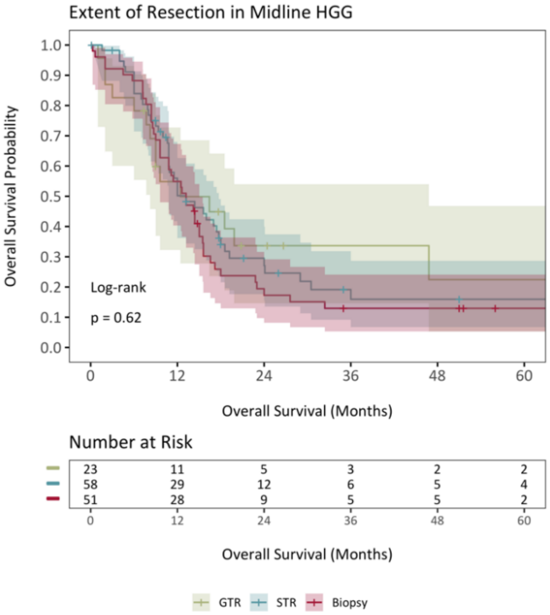

C.)

© 2022 Hatoum R et al. *JAMA Network Open.*
